# Protective effects of sodium hyaluronate nasal spray on murine nasal mucosa via preservation of airway surface liquid and mucociliary clearance

**DOI:** 10.3389/falgy.2026.1866527

**Published:** 2026-06-10

**Authors:** Weizheng Zhao, Jinqi Lang, Yongtai Wang, Liwei Sun, Jiani Li, Cuida Meng, Dongdong Zhu

**Affiliations:** 1 Department of Otolaryngology Head and Neck Surgery, China-Japan Union Hospital of Jilin University, Changchun, China; 2 Jilin Provincial Key Laboratory of Precise Diagnosis and Treatment of Upper Airway Allergic Diseases, Changchun, China; 3 Otolaryngology Head and Neck Surgery Research Center, Changchun, China

**Keywords:** airway surface liquid, hyaluronic acid, inflammatory cytokines, mucociliary clearance, nasal spray, sodium hyaluronate

## Abstract

**Background:**

Saline nasal irrigation is widely used in the management of nasal diseases; however, its inability to replicate the physicochemical properties of airway surface liquid (ASL) may limit its long-term efficacy. We aimed to investigate the protective mechanisms of sodium hyaluronate (hyaluronic acid, HA) nasal spray on the nasal mucosa and to compare its effects with conventional saline.

**Methods:**

BALB/c mice were subjected to intranasal mugwort allergen exposure and treated with either saline or sodium hyaluronate nasal spray for 1–7 days. Behavioral assessments and nasal crusting scores were recorded. Cytokine levels (IL-4, IL-25, IL-33, TSLP) were quantified using ELISA. Histological analyses (H&E, PAS, Alcian Blue) and immunohistochemistry (ZO-1, Occludin) were performed. *Ex vivo* mucociliary clearance (MCC) was assessed under different liquid environments.

**Results:**

HA treatment significantly reduced nasal scratching and attenuated crust formation following prolonged allergen exposure. This was accompanied by suppression of Th2 cytokines and epithelial-derived alarmins in both nasal mucosa and serum. HA preserved mucus layer thickness and goblet cell density and significantly delayed the decline in MCC. Tight junction protein expression remained unchanged.

**Conclusion:**

Sodium hyaluronate nasal spray confers mucosal protection primarily through preservation of ASL homeostasis and MCC function rather than modulation of epithelial tight junctions. These findings highlight HA as a promising alternative to saline for long-term management of nasal mucosal disorders.

## 1 Introduction

Upper airway inflammatory diseases, including allergic rhinitis and chronic rhinosinusitis, are prevalent disorders that substantially impair quality of life and impose significant socioeconomic burdens (1). Nasal irrigation and moisturizing therapy remain fundamental components of conservative management and postoperative care in rhinology. Among these approaches, isotonic saline (0.9% sodium chloride) irrigation is widely recommended in international and domestic guidelines because of its safety, accessibility, and ability to mechanically remove allergens and inflammatory mediators (2, 3). However, despite its broad clinical use, increasing evidence suggests that conventional saline irrigation may destory the complex biochemical and biophysical properties of native airway surface liquid (ASL), particularly under inflammatory conditions.

ASL is a highly specialized bilayer system consisting of a mucus layer (ML) and a periciliary layer (PCL), which together maintain epithelial barrier integrity, local hydration, mucociliary clearance (MCC), and innate immune defense (4). Disruption of ASL homeostasis is increasingly recognized as an important event in the pathogenesis of sinonasal inflammatory diseases. Persistent inflammation, epithelial injury, altered mucus rheology, and impaired hydration can lead to MCC dysfunction, thereby perpetuating chronic mucosal inflammation. Importantly, prolonged or excessive saline irrigation may dilute protective components within ASL, fail to adequately restore the viscoelastic and hygroscopic properties required for optimal mucosal function, and potentially impair MCC, particularly in conditions such as allergic rhinitis and atrophic rhinitis. These clinical limitations highlight the need for novel nasal formulations capable of preserving the physiological microenvironment of native ASL while simultaneously supporting epithelial repair and mucosal homeostasis.

Sodium hyaluronate (hyaluronic acid, HA), a naturally occurring glycosaminoglycan and physiological component of extracellular matrices and airway secretions, has emerged as a promising candidate for nasal mucosal applications because of its unique hydrophilic, viscoelastic, mucoadhesive, and reparative properties. Beyond its physicochemical characteristics, HA can regulate inflammatory signaling through interactions with receptors such as cluster of differentiation 44 (CD44) and Toll-like receptors, thereby influencing epithelial repair, oxidative stress, and immune responses (5, 6). Importantly, accumulating evidence indicates that the biological activity of HA is strongly dependent on molecular weight. High-molecular-weight HA (HMW-HA) has been associated with anti-inflammatory, barrier-protective, and mucosal reparative effects, whereas lower molecular weight fragments may exhibit distinct immunomodulatory properties (7, 8).

In recent years, the intranasal application of HA has become an emerging research focus in rhinology. Clinical studies and meta-analyses have demonstrated beneficial effects of HA in chronic rhinosinusitis, allergic rhinitis, dry nose syndrome, and postoperative sinonasal care (9). HA-based nasal therapies have been shown to improve mucosal healing, reduce postoperative adhesions and edema, enhance MCC, alleviate nasal obstruction and rhinorrhea, and improve patient-reported symptoms while maintaining favorable safety profiles (10). In postoperative functional endoscopic sinus surgery (FESS), topical HA significantly reduced synechiae formation and promoted epithelialization in multiple randomized controlled studies (11). Similarly, adjunctive HA therapy in allergic rhinitis improved mucociliary transport and nasal airflow dynamics (12). Experimental studies further demonstrated that HMW-HA can suppress IL-17A-induced inflammatory signaling, reduce reactive oxygen species production, improve epithelial barrier integrity, and modulate mucus rheology in human nasal epithelial models (8). In parallel, HA has also attracted increasing attention as a mucoadhesive biomaterial and drug-delivery platform capable of prolonging nasal residence time and enhancing drug bioavailability, including in emerging nose-to-brain delivery systems (13–15).

Despite these advances, most existing studies have primarily focused on the pharmacological anti-inflammatory effects of HA, or its drug-delivery capacity. Comparatively little attention has been directed toward a clinically relevant but underappreciated question: whether HA-containing formulations can better preserve the physiological microenvironment of ASL and protect nasal mucosa compared with conventional saline-based formulations routinely used in daily clinical practice. In particular, direct evaluation of ASL integrity and MCC dynamics in the context of nasal moisturizing therapy remains limited. Considering that endogenous HA is an important physiological component of ASL and that recent rhinology guidelines increasingly emphasize mucosal barrier preservation and functional rehabilitation, we hypothesized that sodium hyaluronate nasal spray may better maintain ASL integrity and mucociliary function than conventional saline nasal spray.

To test this hypothesis, we combined *in vivo* and *ex vivo* approaches to investigate the effects of sodium hyaluronate nasal spray on mucosal morphology, ASL mucus integrity, and MCC dynamics under repeated allergen exposure conditions. Unlike previous studies primarily centered on drug delivery or isolated anti-inflammatory pharmacological mechanisms, our work directly addresses a common clinical challenge encountered in routine rhinologic practice: the limitations of saline-based nasal care in maintaining long-term mucosal physiological homeostasis. By focusing on preservation of the native ASL microenvironment rather than solely anti-inflammatory efficacy, this study provides a clinically oriented and mechanistically relevant perspective on HA-based nasal therapy. These findings offer new translational evidence supporting the development of more physiologically optimized and functionally restorative therapeutic strategies for inflammatory sinonasal diseases.

## 2 Materials and methods

### 2.1 Experimental animals

Forty-eight female SPF-grade wild-type BALB/c mice, aged 10–12 weeks and weighing 22 ± 2 g, were obtained from Changchun Yisi Experimental Animal Technology Co., Ltd. Mice were housed in ventilated cages within the barrier animal facility at the College of Basic Medical Sciences, Jilin University. The environment was maintained at 23 ± 1 °C with 50%–60% humidity, and mice had free access to standard chow and water. Animals were acclimated for one week prior to experimentation. All experimental procedures were conducted in accordance with animal ethics guidelines and were approved by the Animal Ethics Committee of the College of Basic Medical Sciences, Jilin University. Female mice were selected to minimize inter-animal variability associated with aggressive behavior and territorial stress commonly observed in group-housed male BALB/c mice. In addition, female mice are widely used in allergic nasal inflammation models because of their stable induction of type 2 inflammatory responses.

### 2.2 Experimental groups and interventions

Forty-eight mice were randomly assigned using a random number table to an *in vivo* experimental group (*n* = 36) and an *ex vivo* experimental group (*n* = 12).

**Figure 1:**
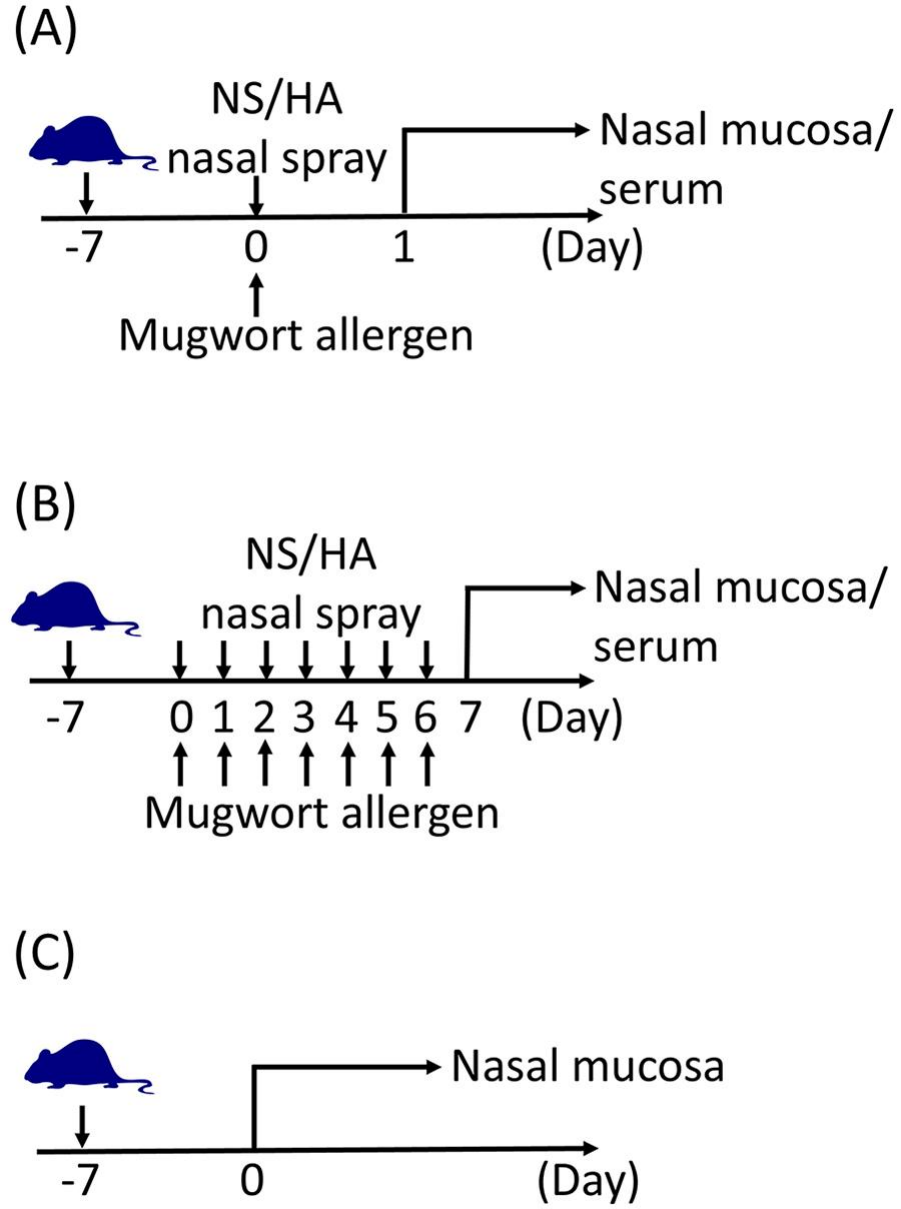
Experimental intervention scheme in mice. **(A)** *In vivo* intervention scheme for the 1-day group. After 7 days of acclimation, mice were exposed to mugwort allergen for 1 day and concurrently received nasal instillations of either sodium hyaluronate nasal spray (HA) or saline (NS) according to group assignment for 1 day. Twenty-four hours after the final administration, mice were euthanized, and nasal mucosa and serum were collected. **(B)** *In vivo* intervention scheme for the 7-day group. Following 7 days of acclimation, mice were exposed to mugwort allergen for 7 days and concurrently received nasal instillations of either sodium hyaluronate nasal spray (HA) or saline (NS) according to group assignment for 7 days. Twenty-four hours after the final administration, mice were euthanized, and nasal mucosa and serum were collected. **(C)** *ex vivo* intervention scheme. After 7 days of acclimation, mice were euthanized and intact nasal mucosae were collected for observation. Samples were exposed to either saline (NS) or sodium hyaluronate nasal spray (HA).

The 36 mice in the *in vivo* group were evenly divided into six subgroups (*n* = 6 per group) based on body weight: 1-day blank control group (CON 1d), 1-day saline model group (NS 1d), 1-day sodium hyaluronate nasal spray intervention group (HA 1d), 7-day blank control group (CON 7d), 7-day saline model group (NS 7d), and 7-day sodium hyaluronate nasal spray intervention group (HA 7d). Mice in the blank control groups (1d and 7d) received no treatment. Mice in the NS and HA groups (1d and 7d) received nasal instillations of the respective agents twice daily, 10 μl per administration (5 μl per nostril), with an 8-h interval between doses (8:30 AM and 4:30 PM). Additionally, 10 min after the first instillation, both groups received a single daily nasal instillation of mugwort allergen extract at 10 mg/mL total protein, 10 μl per dose (5 μl per nostril). The 1d groups were treated for a single day, while the 7d groups received consecutive daily treatments for seven days. Twenty-four hours after the final administration, behavioral characteristics were observed and nasal crusting was assessed, after which the mice were euthanized. Mice were euthanized by exposure to 5% isoflurane in pure oxygen until respiratory arrest and absence of pedal reflex, indicating a surgical plane of anesthesia. Anesthesia was maintained for at least 2 min following cessation of breathing to ensure death, followed by cervical dislocation as a secondary physical euthanasia method. Following euthanasia, 700 μl of peripheral blood was immediately collected from the eyeballs, centrifuged at 4 °C and 1,500 rpm for 15 min, and 300 μl of the supernatant serum was stored at −80 °C for subsequent analyses. Nasal mucosal tissues were collected, with portions wrapped in foil and snap-frozen in liquid nitrogen for inflammatory cytokine assays, and the remaining tissues fixed and decalcified for paraffin embedding and histopathological analysis (Figures 1A,B, Table 1).

**Table 1:** *In vivo* experimental groups and interventions.

Group	Allergen	Treatment	Duration	*n*
CON 1d	None	None	1 day	6
NS 1d	Mugwort	Saline nasal spray	1 day	6
HA 1d	Mugwort	Sodium hyaluronate nasal spray	1 day	6
CON 7d	None	None	7 days	6
NS 7d	Mugwort	Saline nasal spray	7 days	6
HA 7d	Mugwort	Sodium hyaluronate nasal spray	7 days	6

The 12 mice in the *ex vivo* group were evenly divided into two subgroups (*n* = 6 per group) based on body weight: a saline model group (saline group) and a sodium hyaluronate nasal spray control group (HA group). After euthanasia, intact nasal mucosae were immediately collected and immersed in either isotonic saline or sodium hyaluronate nasal spray solutions. Relevant parameters were immediately observed under a microscope (Figure 1C).

### 2.3 Reagents and instruments

0.9% saline (Shandong Qidu Pharmaceutical Co., Ltd., China); Sodium hyaluronate nasal spray (Coolrain, Puyixingrui Shanghai Medical Devices Co., Ltd., China), containing 0.08% high-molecular-weight HA (approximately 1,000–1,200 kDa), sodium chloride, phosphate, and purified water in an isotonic phosphate-buffered aqueous formulation (pH: 6.5–7.5), was designed to provide viscoelastic and hygroscopic properties intended to mimic physiological airway surface liquid conditions; Mugwort allergen (XP47D3A2.5, Greer Labs, USA); ELISA MAX Deluxe Set Mouse IL-4 (431104), ELISA MAX Deluxe Set Mouse IL-25 (447104), LEGENG MAX Mouse IL-33 ELISA Kit (436407), LEGENG MAX Mouse TSLP ELISA Kit (434107) (BioLegend, USA); Hematoxylin and eosin (H&E) staining kit (P0040, Pinuofei Biotechnology Co., Ltd., China); Periodic acid–Schiff (PAS) staining kit (110802015, Changde Bikeman Biotechnology Co., Ltd., China); Alcian Blue staining solution (MM402, Beijing Transgen Biotechnology Co., Ltd., China); Occludin Polyclonal antibody (13409-1-AP), ZO-1 Polyclonal antibody (21773-1-AP) (Proteintech Group, China); UltraSensitive SP immunohistochemistry kit (KIT-9720, Fuzhou Maxim Biotechnology Development Co., Ltd., China); DAB chromogenic kit (BL732A, Beijing Labgic Technology Co., Ltd., China); Anhydrous ethanol and xylene (Sinopharm Chemical Reagent Co., Ltd., China); Refrigerated high-speed centrifuge and microplate reader (Thermo Scientific, USA); Optical microscope (Olympus, Japan); Fully automated rapid tissue grinder (Shanghai Jingxin Industrial Development Co., Ltd., China).

### 2.4 Behavioral assessment in mice

Twenty-four hours after the final administration, *in vivo* mice were observed for 30 min to record nasal scratching behavior. Nasal scratching was scored according to the following criteria: 1 point for mild scratching (≤3 times) and 2 points for continuous facial scratching (> 3 times). The total nasal scratching score for each mouse was then calculated.

### 2.5 Local symptom scoring in mice

Twenty-four hours after the final administration, photographs were taken to document the severity of nasal crusting in the *in vivo* experimental mice. Scoring was assigned as follows: 0 points for no crusting, 1 point for slight crusting without noticeable color change, and 2 points for extensive crusting accompanied by marked color changes of the nose.

### 2.6 Assessment of inflammatory cytokines in mouse nasal Mucosa and Serum

Frozen nasal mucosa tissues were weighed and homogenized in cold phosphate-buffered saline (PBS) (4 °C) at a 1:9 tissue-to-buffer ratio using bead-containing homogenization tubes. Homogenization was performed at 60 Hz for 2 min at 4 °C, repeated twice. The homogenates were centrifuged at 12,000 g for 15 min at 4 °C. Three hundred microliters of the supernatant were collected and diluted 1:2 with an equal volume of PBS, then stored at 4 °C for further use. Total protein concentrations were determined using the bicinchoninic acid (BCA) assay to ensure uniform protein content across samples. Serum samples stored at −80 °C were thawed and diluted 1:4 with saline, then stored at 4 °C until use. Levels of interleukin-4 (IL-4), interleukin-25 (IL-25, also termed IL-17E), interleukin-33 (IL-33) IL-33, and thymic stromal lymphopoietin (TSLP) in nasal mucosa and serum were measured strictly following the instructions provided with the respective ELISA kits. Absorbance at 450 nm was measured using a microplate reader, with readings at 570 nm taken simultaneously for background correction. Cytokine concentrations were calculated by fitting a four-parameter logistic curve to the standard curve absorbance values.

### 2.7 Histopathological analysis of mouse nasal mucosa

After euthanasia, portions of nasal mucosa from the *in vivo* experimental mice were snap-frozen for subsequent analyses, and the remaining nasal tissues were fixed in 4% formalin for 72 h and decalcified in 5% formic acid for 48 h. Following graded ethanol dehydration, xylene clearing, and paraffin embedding, continuous sections of 5 μm thickness were prepared and baked at 60 °C for 3 h for subsequent use.

#### 2.7.1 Hematoxylin and eosin (H&E) staining

Sections were deparaffinized in xylene, rehydrated through a graded ethanol series, and hydrated in distilled water. They were then stained with hematoxylin for 5 min, differentiated in differentiation solution for 15 s, and rinsed under running water for bluing, followed by eosin staining for 5 min. Sections were dehydrated through a graded ethanol series, cleared in xylene, and mounted with neutral resin. Five high-power fields at the same anatomical site were selected per mouse under a microscope. Nasal epithelial damage, ciliary morphology, and inflammatory cell infiltration were evaluated according to the mucosal injury scoring criteria described by Ponikau et al., (16).

#### 2.7.2 Periodic acid–schiff (PAS) staining

Sections were deparaffinized in xylene, rehydrated through a graded ethanol series, and hydrated in distilled water. They were then treated with periodic acid in the dark for 8 min, followed by staining with Schiff reagent in the dark for 10 min. Sections were counterstained with hematoxylin for 2 min, differentiated in 1% hydrochloric acid in ethanol for 10 s, and rinsed under running water for 15 min for bluing. Subsequent dehydration, clearing, and mounting procedures were performed as described for H&E staining. Five high-power fields at the same anatomical site were selected per mouse under a microscope. The number of mucus-secreting cells within equivalent areas was counted, and the mean value was calculated to assess goblet cell secretory activity.

#### 2.7.3 Alcian blue staining

Sections were deparaffinized in xylene, rehydrated through a graded ethanol series, and hydrated in distilled water. They were then stained with Alcian Blue solution for 10 min and rinsed under running tap water for 10 min. Subsequent dehydration, clearing, and mounting procedures were performed as described for H&E staining. Five high-power fields at the same anatomical site were selected per mouse under a microscope. The average thickness of the mucus layer was measured and calculated to assess the distribution of surface mucus in the nasal mucosa.

#### 2.7.4 Immunohistochemistry (IHC) staining

Sections were deparaffinized in xylene, rehydrated through a graded ethanol series, and hydrated in distilled water, followed by three washes with PBS for 3 min each. Antigen retrieval was performed in ethylenediaminetetraacetic acid (EDTA) retrieval solution using a microwave at high power for 2 min, then low power for 10 min, followed by cooling in water for 20 min and three PBS washes for 3 min each. Endogenous peroxidase activity was blocked by incubation with a blocking reagent for 10 min, followed by three PBS washes for 3 min each. Sections were incubated with a nonspecific blocking reagent for 5 min. After discarding the blocking solution, primary antibodies (Occludin, 1:100; zonula occludens-1, 1:50) were applied and incubated overnight at 4 °C, followed by three PBS washes for 3 min each. Sections were incubated with secondary antibodies at 37 °C for 30 min, followed by three PBS washes for 3 min each. Streptavidin–peroxidase complex was applied for 10 min, followed by three PBS washes for 3 min each. Visualization was achieved using 3,3′-diaminobenzidine (DAB) substrate for 10 min, stopped with PBS, and sections were counterstained with hematoxylin, differentiated, blued, dehydrated, cleared, and mounted. Five high-power fields at the same anatomical site were selected per mouse under a microscope. H-SCOREs for Occludin and zonula occludens-1 (ZO-1) were calculated to evaluate the expression of tight junction proteins.

All histopathological (H&E, PAS, and Alcian Blue staining), immunohistochemical (ZO-1 and Occludin staining) were performed in a blinded manner. Tissue sections were coded using random identifiers prior to analysis, and all scoring and quantitative assessments were conducted without knowledge of group allocation. Histopathological injury scoring, goblet cell quantification, mucus layer thickness measurements, and immunohistochemical H-SCORE analyses were independently evaluated by two investigators. Discrepancies between the two investigators were resolved by independent evaluation from a third, more experienced researcher, followed by consensus integration of all three assessments. Quantitative image analyses were performed using ImageJ software under identical image acquisition parameters and threshold settings across all groups to ensure analytical consistency and reproducibility.

### 2.8 Functional assessment of mouse nasal mucosa

After euthanasia, intact nasal mucosal tissues from *ex vivo* experimental mice were rapidly harvested, carefully dissected, and immediately mounted flat on glass slides with the epithelial surface facing upward. Tissue preparation was performed with particular care to preserve the native mucus layer and avoid mechanical disruption of the ciliated epithelium. Subsequently, 300 μL of pre-warmed (37 °C) isotonic saline or sodium hyaluronate nasal spray solution was gently applied to fully cover the mucosal surface. Mucociliary transport activity and ciliary beating were continuously observed and recorded using an optical microscope equipped with a high-speed digital camera (Olympus, Japan) at 200× magnification. Video recordings were obtained at predefined time points (0, 10, 20, 30, 40, 50, and 60 min after liquid application). Nasal mucosal tissues were maintained at 37 °C throughout imaging. MCC velocity was defined as the mean particle displacement over time and quantified by tracking the displacement of mucus particles and debris naturally present on the epithelial surface. For each field, five particles were tracked using ImageJ software. Three independent fields of view from comparable anatomical regions were randomly selected for each specimen, and the mean MCC velocity was calculated as particle displacement distance divided by time and expressed as μm/s. To improve reproducibility and minimize observational bias, all recordings, MCC measurements, and image analyses were performed in a blinded manner by two investigators, and triplicate measurements were obtained for each time point.

### 2.9 Statistical analysis

Experimental data were organized using Microsoft Excel and image analysis was performed with ImageJ. Statistical analyses were conducted using IBM SPSS 27.0 and GraphPad Prism 10. Normality of data distribution was assessed using the Shapiro–Wilk test prior to statistical comparisons. Normally distributed continuous variables are expressed as mean ± standard deviation. Comparisons between two groups were performed using independent-samples *t*-tests, while comparisons among multiple groups were conducted using one-way analysis of variance (ANOVA). Tukey's multiple comparisons test was used for *post hoc* analysis following one-way ANOVA. Continuous variables not following a normal distribution are presented as median (interquartile range). Comparisons between two groups were performed using the Mann–Whitney (*U*) test, and comparisons among multiple groups were conducted using the Kruskal–Wallis (*H*) test. Dunn's multiple comparisons test was used for *post hoc* analysis following the Kruskal–Wallis test. Categorical variables are expressed as counts (percentages), and comparisons between groups were performed using the *χ*^2^ test. *P* < 0.05 was considered statistically significant. Sample size (*n* = 6 per group) was determined *a priori* based on previous murine studies and the overall experimental design.

## 3 Results

### 3.1 Behavioral and local symptom changes in mice

**Figure 2:**
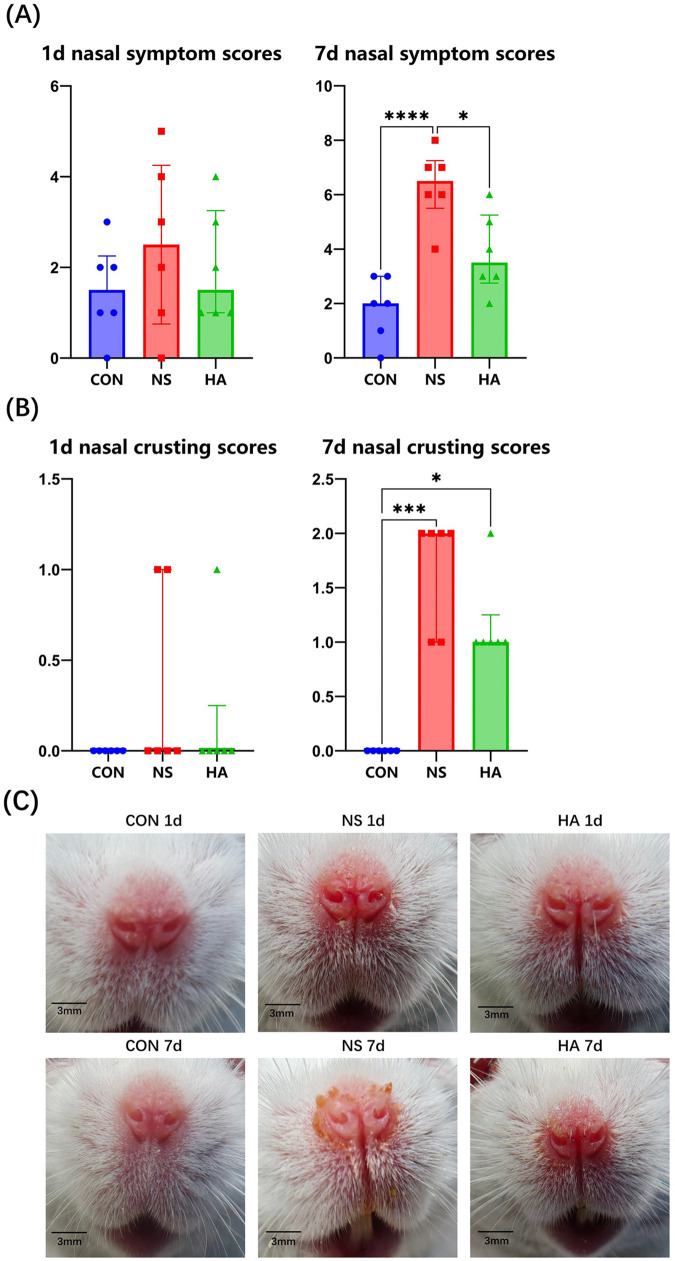
Behavioral and local symptom outcomes in mice. **(A)** Nasal symptom scores of mice in each group after 1 and 7 days of intervention (*n* = 6 per group). **(B)** Nasal crusting scores of mice in each group after 1 and 7 days of intervention (*n* = 6 per group). **(C)** Representative images of nasal crusting in mice from each group after 1 and 7 days of intervention. CON, control group; NS, saline model group; HA, sodium hyaluronate nasal spray group. *P < 0.05, **P < 0.01, ***P < 0.001.

Scratch scores in the CON 1d, NS 1d, and HA 1d groups were 1.50 ± 1.05, 2.50 ± 1.87, and 2.00 ± 1.27, respectively, with no significant differences observed among the groups (*F* = 0.72, *P* = 0.5002). Scratch scores in the CON 7d, NS 7d, and HA 7d groups were 1.83 ± 1.17, 6.33 ± 1.37, and 3.83 ± 1.47, respectively. Mice in the NS 7d group exhibited significantly more severe scratching behavior compared with the CON 7d and HA 7d groups (*F* = 16.94, *P* < 0.0001), whereas no significant difference was observed between the CON 7d and HA 7d groups (*P* = 0.515) (Figure 2A).

Nasal crusting scores in the CON 1d, NS 1d, and HA 1d groups were 0.0 (0.0), 0.0 (1.0), and 0.0 (0.25), respectively, with no significant differences in severity observed among the groups (*H* = 2.267, *P* = 0.7353). Nasal crusting scores in the CON 7d, NS 7d, and HA 7d groups were 0.0 (0.0), 2.0 (1.0), and 1.0 (0.25), respectively. Mice in both the NS 7d and HA 7d groups exhibited significantly more severe nasal crusting than those in the CON 7d group (H = 13.91, *P* < 0.0001), whereas no significant difference was observed between the NS 7d and HA 7d groups (*P* = 0.903) (Figures 2B,C).

After 1 day of intervention, no significant differences were observed in scratching scores or nasal crusting among the CON, NS, and HA groups, suggesting that short-term allergen exposure and reagent administration had minimal effects on nasal symptoms in mice. After 7 days of continuous intervention, scratching scores in the NS group were significantly higher than those in the CON and HA groups, whereas no significant difference was observed between the CON and HA groups. Nasal crusting scores in both the NS and HA groups were elevated compared with the CON group, with the NS group exhibiting the greatest severity. These results indicate that prolonged allergen exposure substantially exacerbates nasal symptoms in mice, while sodium hyaluronate nasal spray effectively mitigates allergen-induced scratching behavior and improves local nasal symptoms.

### 3.2 Levels of cytokines in mouse nasal mucosa and serum

#### 3.2.1 Nasal mucosal and serum IL-4 levels

Nasal mucosal IL-4 levels in the CON 1d, NS 1d, and HA 1d groups were 40.19 ± 1.12 pg/mL, 41.42 ± 1.20 pg/mL, and 39.57 ± 1.40 pg/mL, respectively, with no significant differences observed among the groups (*F* = 3.43, *P* = 0.0596). Serum IL-4 levels in the three groups were 101.2 ± 6.63 pg/mL, 109.3 ± 12.22 pg/mL, and 110.6 ± 10.01 pg/mL, respectively, with no significant differences observed (*F* = 1.613, *P* = 0.2284).

**Figure 3:**
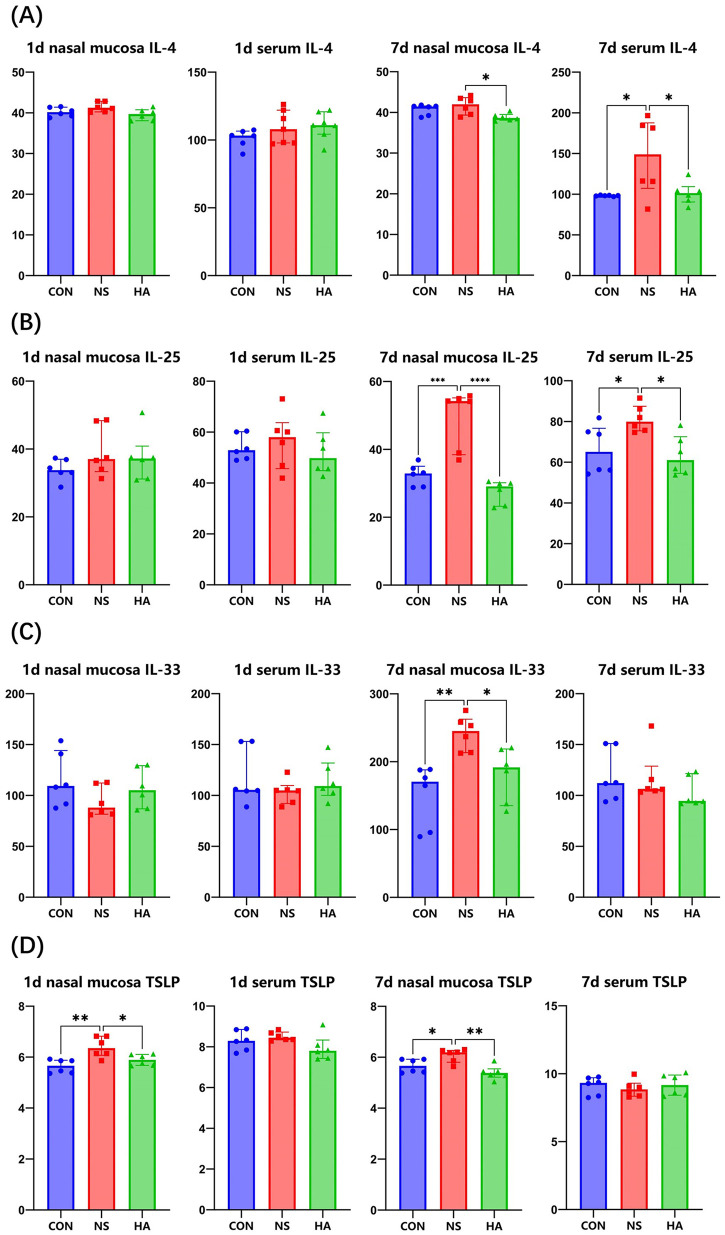
Nasal mucosal and serum cytokine levels in mice. **(A)** Nasal mucosal and serum IL-4 levels in each group after 1 and 7 days of intervention (*n* = 6 per group). **(B)** Nasal mucosal and serum IL-25 levels in each group after 1 and 7 days of intervention (*n* = 6 per group). **(C)** Nasal mucosal and serum IL-33 levels in each group after 1 and 7 days of intervention (*n* = 6 per group). **(D)** Nasal mucosal and serum TSLP levels in each group after 1 and 7 days of intervention (*n* = 6 per group). CON, control group; NS, normal saline model group; HA, sodium hyaluronate nasal spray intervention group. *P < 0.05, **P < 0.01, ***P < 0.001.

After 7 days of intervention, nasal mucosal IL-4 levels in the CON 7d, NS 7d, and HA 7d groups were 40.69 ± 1.32 pg/mL, 41.64 ± 2.17 pg/mL, and 38.80 ± 0.89 pg/mL, respectively. IL-4 levels in the NS 7d group were significantly higher than those in the HA 7d group (*F* = 5.819, *P* = 0.0194). Serum IL-4 levels in the CON 7d, NS 7d, and HA 7d groups were 98.14 ± 0.63 pg/mL, 146.0 ± 47.42 pg/mL, and 101.2 ± 13.65 pg/mL, respectively. Serum IL-4 levels in the NS 7d group were significantly higher than those in the CON 7d and HA 7d groups (*F* = 5.313, *P* = 0.0180) (Figure 3A).

#### 3.2.2 Nasal mucosal and Serum IL-25 Levels

Nasal mucosal IL-25 levels in the CON 1d, NS 1d, and HA 1d groups were 33.92 ± 3.11, 39.38 ± 7.36, and 37.47 ± 7.20 pg/mL, respectively, with no significant differences observed among the groups (*F* = 1.193, *P* = 0.3304). Serum IL-25 levels in the three groups were 54.12 ± 5.02, 56.40 ± 11.03, and 52.06 ± 9.35 pg/mL, respectively, with no significant differences observed (*F* = 0.3602, *P* = 0.7022).

After 7 days of intervention, nasal mucosal IL-25 levels in the CON 7d, NS 7d, and HA 7d groups were 32.46 ± 3.15, 49.15 ± 8.75, and 27.48 ± 3.49 pg/mL, respectively. IL-25 levels in the NS 7d group were significantly higher than those in both the CON 7d and HA 7d groups (*F* = 23.48, *P* < 0.0001). Serum IL-25 levels in the CON 7d, NS 7d, and HA 7d groups were 66.20 ± 11.98, 81.29 ± 6.57, and 63.22 ± 9.83 pg/mL, respectively. Serum IL-25 levels in the NS 7d group were significantly higher than those in the CON 7d and HA 7d groups (*F* = 5.959, *P* = 0.0125) (Figure 3B).

#### 3.2.3 Nasal mucosal and Serum IL-33 Levels

Nasal mucosal IL-33 levels in the CON 1d, NS 1d, and HA 1d groups were 115.4 ± 26.61, 93.91 ± 14.89, and 107.0 ± 19.54 pg/mL, respectively, with no significant differences observed among the groups (*F* = 1.608, *P* = 0.2330). Serum IL-33 levels in the three groups were 118.4 ± 27.62, 103.3 ± 11.88, and 114.6 ± 19.65 pg/mL, respectively, with no significant differences observed (*F* = 0.8567, *P* = 0.4443).

After 7 days of intervention, nasal mucosal IL-33 levels in the CON 7d, NS 7d, and HA 7d groups were 150.5 ± 45.67, 241.7 ± 25.42, and 181.1 ± 39.94 pg/mL, respectively. IL-33 levels in the NS 7d group were significantly higher than those in both the CON 7d and HA 7d groups (*F* = 8.964, *P* = 0.0027). Serum IL-33 levels in the CON 7d, NS 7d, and HA 7d groups were 119.5 ± 25.51, 117.4 ± 25.28, and 103.0 ± 14.83 pg/mL, respectively, with no significant differences observed (*F* = 0.9642, *P* = 0.4037) (Figure 3C).

#### 3.2.4 Nasal mucosal and Serum TSLP levels

Nasal mucosal TSLP levels in the CON 1d, NS 1d, and HA 1d groups were 5.64 ± 0.27, 6.39 ± 0.40, and 5.89 ± 0.21 pg/mL, respectively. TSLP Levels in the NS 1d group were significantly higher than those in both the CON 1d and HA 1d groups (*F* = 9.470, *P* = 0.0022). Serum TSLP levels in the three groups were 8.30 ± 0.50, 8.51 ± 0.22, and 7.93 ± 0.62 pg/mL, respectively, with no significant differences observed among groups (*F* = 2.026, *P* = 0.1387).

After 7 days of intervention, nasal mucosal TSLP levels in the CON 7d, NS 7d, and HA 7d groups were 5.66 ± 0.27, 6.07 ± 0.26, and 5.40 ± 0.27 pg/mL, respectively. TSLP Levels in the NS 7d group were significantly higher than those in the CON 7d and HA 7d groups (*F* = 9.848, *P* = 0.0019). Serum TSLP levels in the CON 7d, NS 7d, and HA 7d groups were 9.11 ± 0.66, 8.89 ± 0.61, and 9.17 ± 0.80 pg/mL, respectively, with no significant differences observed (*F* = 0.2621, *P* = 0.7729) (Figure 3D).

These findings suggest that sodium hyaluronate nasal spray effectively reduces allergen-induced expression of local Th2-type cytokines and epithelial-derived alarmins in the nasal mucosa, and also mitigates the elevation of systemic inflammatory cytokines in serum. TSLP responds more rapidly to allergen stimulation, showing increased expression after short-term exposure, whereas other cytokines, such as IL-4, IL-25, and IL-33, require repeated exposure to exhibit upregulation.

### 3.3 Histopathological changes in mouse nasal mucosa

#### 3.3.1 Hematoxylin and eosin (HE) staining

**Figure 4:**
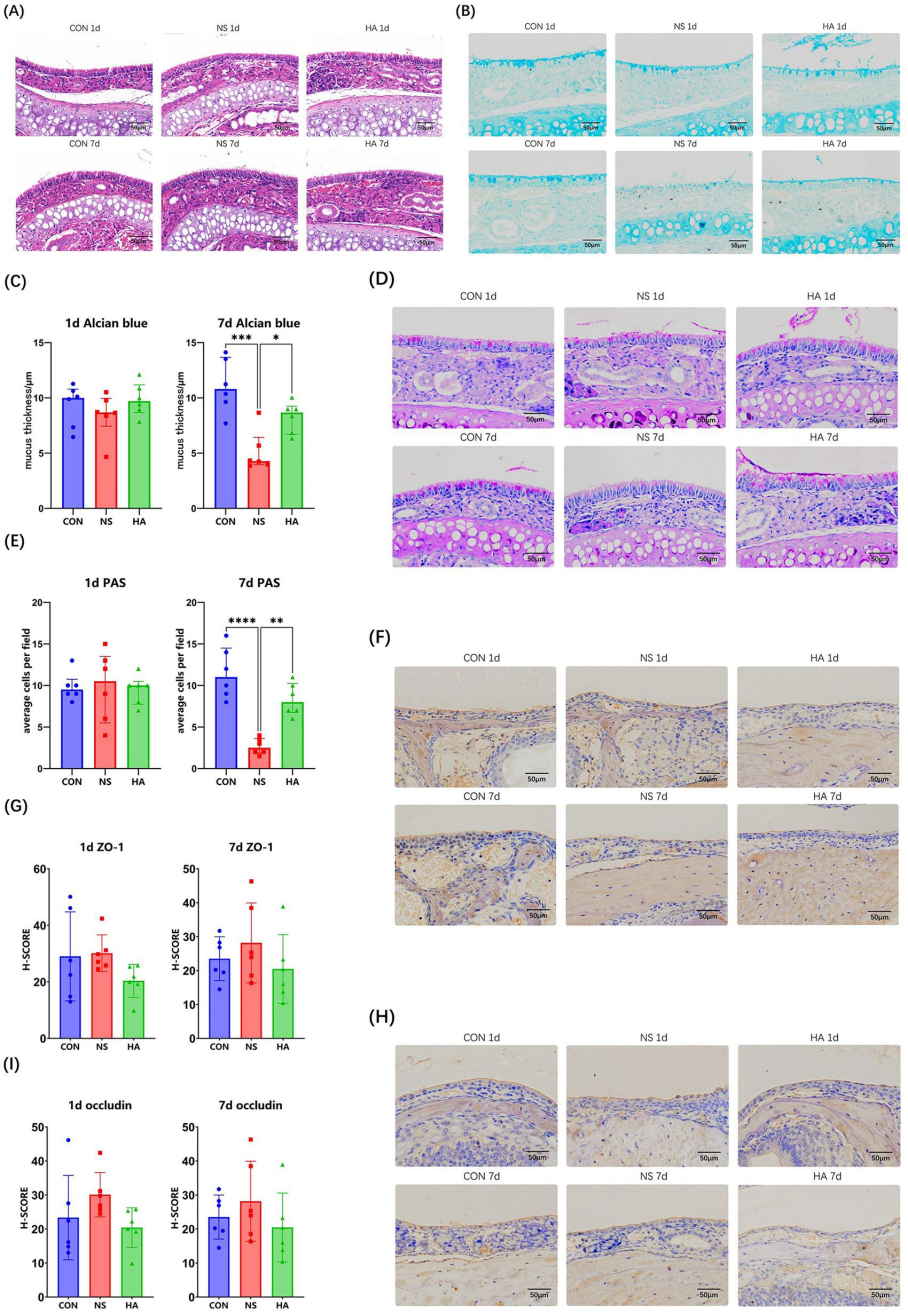
Histopathological changes in mouse nasal Mucosa. **(A)** Representative hematoxylin and eosin (HE) staining images of nasal mucosa from each group at 1 and 7 days. **(B)** Representative Alcian Blue-stained images of nasal mucosa from each group at 1 and 7 days. **(C)** Average mucus layer thickness measured from Alcian Blue-stained nasal mucosa in each group at 1 and 7 days (*n* = 6 per group). **(D)** Representative periodic acid–Schiff (PAS) staining images of nasal mucosa from each group at 1 and 7 days. **(E)** Average number of goblet cells per high-power field in PAS-stained nasal mucosa at 1 and 7 days (*n* = 6 per group). **(F)** Representative immunohistochemical staining images of ZO-1 in nasal mucosa at 1 and 7 days. **(G)** Average H-SCORE of ZO-1 immunostaining in nasal mucosa at 1 and 7 days (*n* = 6 per group). **(H)** Representative immunohistochemical staining images of Occludin in nasal mucosa at 1 and 7 days. **(I)** Average H-SCORE of Occludin immunostaining in nasal mucosa at 1 and 7 days (*n* = 6 per group). CON, control group; NS, normal saline model group; HA, sodium hyaluronate nasal spray intervention group. *P < 0.05, **P < 0.01, ***P < 0.001.

HE-stained sections were evaluated using the mucosal injury scoring system described by Ponikau in 2003. For each group, 30 high-power fields (HPFs) per mouse were assessed to determine the degree of mucosal damage, and the proportion of fields with different injury grades was used to quantify mucosal damage across groups. In the CON 1d, NS 1d, HA 1d, and CON 7d groups, the nasal mucosa showed no apparent damage. No typical epithelial detachment or ciliary loss was observed. Cilia were well-formed and regularly arranged, the epithelial barrier remained intact, and no significant inflammatory cell infiltration or mucosal edema was detected. Most fields were classified as grade 0 injury, with occasional grade 1 lesions. In the HA 7d group, ciliary arrangement was slightly less regular, with rare ciliary loss. Submucosal inflammatory infiltration and mucosal edema were minimal, and grade 1 injury was observed in a small number of HPFs. In the NS 7d group, ciliary loss was observed on portions of the nasal mucosa, accompanied by mild inflammatory cell infiltration. Grade 1 injury was common, with occasional grade 2 lesions. Overall, no significant differences in mucosal injury were observed among the CON 1d, NS 1d, and HA 1d groups. The NS 7d group exhibited significantly greater nasal mucosal damage compared to both the HA 7d and CON 7d groups (Figure 4A, Table 2). These results indicate that prolonged allergen exposure can induce nasal epithelial damage, whereas sodium hyaluronate nasal spray effectively mitigates allergen-induced structural disruption of the nasal mucosa.

**Table 2:** HE mucosal injury in each group.

Histological Injury Grade	CON 1d	NS1d	HA 1d	CON 7d	NS 7d	HA 7d
Proportion of Grade 0 Injury (%)	96.6	96.6	93.3	96.6	80	90
Proportion of Grade 1 Injury (%)	3.34	3.34	6.67	3.34	13.33	10
Proportion of Grade 2 Injury (%)	0	0	0	0	6.67	0
Proportion of Grade 3 Injury (%)	0	0	0	0	0	0

#### 3.3.2 Alcian blue staining

Alcian Blue-stained sections were used to measure the thickness of the mucosal surface mucus layer in each high-power field (HPF). The average thickness per mouse was calculated to evaluate the distribution and integrity of airway surface liquid across groups. No significant differences in mucus layer thickness were observed among the CON 1d, NS 1d, HA 1d, CON 7d, and HA 7d groups (*P* > 0.05). Mucus layer thickness in the NS 7d group was significantly lower than in the CON 7d and HA 7d groups (*F* = 14.24, *P* = 0.0003), indicating reduced epithelial mucus content and exacerbated mucus layer disruption in the NS 7d group (Figures 4B,C). These findings suggest that sodium hyaluronate nasal spray effectively preserves mucus layer thickness and maintains airway surface liquid integrity in mice following allergen exposure.

#### 3.3.3 Periodic acid–schiff (PAS) staining

PAS staining was performed by counting the number of mucin-secreting cells within the same area in each high-power field (HPF). The average number of cells per mouse was calculated to compare goblet cell secretory activity across groups. No significant differences in the average number of secretory cells were observed among the CON 1d, NS 1d, HA 1d, CON 7d, and HA 7d groups (*P* > 0.05). The NS 7d group exhibited a significantly lower number of secretory cells compared with the CON 7d and HA 7d groups (*F* = 25.15, *P* < 0.0001), indicating reduced epithelial mucin secretion and a decrease in goblet cell numbers (Figures 4D,E). These findings suggest that sodium hyaluronate nasal spray effectively preserves goblet cell secretory function in mouse nasal mucosa following allergen exposure, reducing the loss of mucin-secreting cells.

#### 3.3.4 Immunohistochemical (IHC) staining

Immunohistochemical staining was performed to calculate the average H-SCOREs for Occludin and ZO-1 in high-power fields (HPFs) of each group. This analysis was used to evaluate and compare the expression of airway epithelial tight junction proteins across groups (17, 18). The results showed no significant differences in Occludin and ZO-1 H-SCOREs among the groups, including CON 1d, NS 1d, HA 1d, CON 7d, NS 7d, and HA 7d (*P* > 0.05). This indicates that expression of epithelial tight junction proteins in the nasal mucosa was comparable across groups, with no evidence of tight junction disruption (Figures 4F–I). These findings suggest that neither allergen exposure nor pharmacological intervention significantly affected the expression of epithelial tight junction proteins in the nasal mucosa. The protective effect of sodium hyaluronate nasal spray appears to be mediated primarily by maintaining airway surface liquid homeostasis rather than by modulating tight junction protein expression.

### 3.4 Assessment of nasal mucosal function in mice

The average MCC rate of nasal mucosa was measured in two *ex vivo* experimental groups of mice, with tissues immersed in either normal saline or sodium hyaluronate nasal spray. The temporal changes in clearance rates were recorded to compare the protective effects of the two solutions on nasal mucosal clearance function. The results showed that the initial MCC rate was identical in both groups at 20 μm/s. At 10 min, the rate decreased to 5 μm/s in the saline group and 15 μm/s in the hyaluronate group. At 20 min, the saline group's MCC fell below 3 μm/s, indicating minimal clearance, whereas the hyaluronate group maintained 10 μm/s. At 30 min, the saline group's MCC dropped below 1 μm/s, essentially losing clearance ability, while the hyaluronate group remained at 8 μm/s. Subsequently, the hyaluronate group maintained 5 μm/s at 40 min, fell below 3 μm/s at 50 min, and dropped below 1 μm/s at 60 min, indicating near-complete loss of clearance. These findings indicate that sodium hyaluronate nasal spray significantly delays the decline of MCC in mouse nasal mucosa compared with saline. It prolongs the maintenance of MCC, preserves the airway surface liquid microenvironment, and supports normal mucosal clearance function (Supplementary Material: Video S1).

## 4 Discussion

This study provides mechanistic evidence that sodium hyaluronate nasal spray protects the nasal mucosa primarily by preserving ASL integrity and sustaining MCC, rather than by directly modulating epithelial tight junctions.

ASL is a bilayer fluid structure widely present on the airway surfaces of mammals, consisting of an upper mucus layer and a lower periciliary layer. This bilayer system, regulated by precise secretion mechanisms and coordinated ciliary motion, mediates MCC and plays a critical role in maintaining the integrity of the airway epithelial barrier (19). The ASL constitutes a dynamic and tightly regulated interface that integrates biophysical and immunological functions. In recent years, as understanding of the ASL bilayer has deepened, increasing attention has been paid to its alterations during the pathogenesis of upper airway diseases, such as chronic rhinosinusitis and allergic rhinitis. Evidence indicates that the onset of nasal inflammatory diseases is frequently associated with structural disruption and functional impairment of the ASL (20). Its disruption not only impairs MCC but also amplifies epithelial exposure to allergens and irritants, thereby promoting type 2 inflammation. Therefore restoring normal ASL architecture and function is crucial for the treatment and prevention of nasal disorders. In this context, our findings position ASL preservation as a central therapeutic target.

Nasal irrigation with isotonic saline (0.9% sodium chloride) has become an essential approach for perioperative management and routine care of nasal inflammatory diseases, and it is widely applied in clinical practice (2, 3, 21). However, with increasing understanding of ASL, particularly nasal mucosal ASL, the limitations of using saline alone for local nasal application have become increasingly apparent. Because the composition of saline differs significantly from that of the nasal mucosal ASL (22), its local application can temporarily relieve nasal symptoms in some patients; however, long-term use may disrupt the normal structure and clearance function of the nasal ASL, thereby adversely affecting patient outcomes (23), Clinically, we have observed that with prolonged use of saline nasal irrigation, patients may experience worsening nasal dryness and related symptoms, which is particularly evident in individuals with allergic rhinitis or atrophic rhinitis (24). Although saline application can facilitate the clearance of purulent secretions and sanguineous crusts in patients undergoing perioperative management of acute or chronic rhinosinusitis, it is clearly unsuitable for the treatment of chronic non-suppurative inflammation, such as allergic rhinitis or atrophic rhinitis, or for routine maintenance of normal nasal mucosa. Therefore, there is an urgent need for a novel nasal spray to replace conventional saline for adjunctive treatment of nasal disorders and routine nasal care, which can alleviate symptoms while preserving the normal structure and function of the airway surface liquid.

HA is a natural polysaccharide widely distributed in human connective tissues, skin, and body fluids, where it plays critical roles in hydration, lubrication, and tissue repair (25). It has been extensively applied in routine skin care and as an adjunct therapy for related disorders (26, 27). Considering that ASL physiologically contains a certain amount of HA, and in accordance with the latest domestic guideline recommendations (2), we selected a novel nasal spray formulation containing 0.08% sodium hyaluronate, sodium chloride, phosphate, and purified water, with a pH of 6.5–7.5. The formulation employs sterile patented packaging to maintain sterility after opening. Importantly, the HA used in this formulation belongs to the high-molecular-weight HA (HMW-HA) range (approximately 1,000–1,200 kDa), which is generally considered to possess favorable hydrophilic, viscoelastic, and barrier-protective properties. In contrast to low-molecular-weight HA fragments, which may activate Toll-like receptor-mediated pro-inflammatory signaling pathways, HMW-HA has been consistently associated with anti-inflammatory activity, epithelial protection, maintenance of tissue hydration, and stabilization of mucosal viscoelasticity. Notably, the rheological characteristics of this formulation provide moderate viscosity and hygroscopicity that more closely resemble the composition and biophysical behavior of physiological nasal secretions and native ASL. This novel HA-containing nasal spray was therefore used to compare its effects with saline nasal spray and to investigate its protective effects and underlying mechanisms on murine nasal mucosa.

To investigate the effects and underlying mechanisms of sodium hyaluronate nasal spray on murine nasal mucosa, we conducted both *in vivo* and *ex vivo* experiments. Compared with saline, HA demonstrated a superior capacity to preserve nasal mucosal homeostasis, particularly under conditions of repeated allergen exposure. This protective effect was primarily reflected in the maintenance of ASL integrity and MCC function, rather than direct modulation of epithelial barrier structures. Notably, HA treatment effectively maintained mucus layer thickness and goblet cell secretory function following prolonged allergen stimulation. This effect is likely attributable to the intrinsic viscoelastic and hygroscopic properties of HA, which stabilize the periciliary microenvironment and prevent dehydration-induced ciliary dysfunction. Consistent with this, *ex vivo* experiments further demonstrated that HA significantly delayed the decline of MCC activity compared with saline, indicating that HA prolongs functional ciliary activity by sustaining optimal ASL rheology. Together, these findings suggest that HA exerts a critical biophysical role in preserving mucosal surface dynamics. Importantly, HA administration significantly attenuated the expression of epithelial-derived alarmins, including TSLP, IL-25, and IL-33, which are key initiators of type 2 immune responses. This inhibitory effect is unlikely to be solely explained by direct immunomodulatory actions of HA. Instead, it is more plausibly attributed to reduced epithelial stress and decreased allergen contact, secondary to preserved MCC function. In this context, TSLP, as a highly sensitive epithelial alarmin, can rapidly respond to environmental stimuli such as allergens, temperature changes, and irritants (28, 29). Interestingly, immunohistochemical analysis revealed no significant differences in the expression of tight junction proteins (ZO-1 and Occludin) between HA and saline-treated groups. The absence of detectable changes in ZO-1 and Occludin expression may reflect the relatively early stage of mucosal injury in this model, during which functional ASL alterations precede overt epithelial barrier disruption. This also indicates that epithelial barrier integrity was not the primary site of HA-mediated protection. Such findings contrast with traditional paradigms that emphasize barrier repair as a central therapeutic target and instead support a model in which ASL dysfunction precedes overt epithelial barrier disruption. Accordingly, the preservation of ASL function appears to be a more critical determinant of early mucosal protection.

Under short-term allergen exposure (1 day), no significant structural or functional damage to the nasal mucosa was observed, and mucosal surface liquid distribution and goblet cell secretion remained largely intact. Consequently, HA treatment did not confer a pronounced advantage over saline in most parameters, except for a reduction in TSLP levels. However, with repeated allergen exposure, the nasal mucosa exhibited progressive structural deterioration, including thinning of the ASL layer, disorganization of ciliary architecture, and partial ciliary loss. Under these conditions, saline failed to prevent mucosal damage and was associated with enhanced systemic type 2 responses, as indicated by elevated serum IL-4 and IL-25 levels. In contrast, HA treatment effectively mitigated these pathological changes, preserving ASL thickness, maintaining goblet cell function, and reducing local inflammatory cytokine levels (IL-4, IL-25, IL-33, and TSLP). Previous studies on HA have predominantly focused on its pharmacological and immunomodulatory properties, including the regulation of dendritic cell maturation and suppression of Th2 responses through reduced IL-4 and IL-13 production (30). Additionally, HA interactions with receptors such as CD44 and TLR4 have been shown to modulate downstream NF-κB and MAPK signaling pathways, thereby inhibiting pro-inflammatory cytokine transcription (31, 32).While these biochemical mechanisms are well established, our findings suggest that this specific HMW-HA (approximately 1,000–1,200 kDa) formulation may exert an additional and clinically meaningful protective role by preserving ASL integrity and maintaining mucociliary MCC dynamics, thereby supporting the physical barrier function of the nasal mucosa. These observations extend the current understanding of HA from a predominantly pharmacologically active biomaterial to a physiologically restorative component capable of stabilizing the nasal mucosal microenvironment under inflammatory conditions. Specifically, HA-mediated stabilization of ASL and preservation of MCC function appear to play a central role in limiting epithelial activation and subsequent inflammation. This distinction is clinically relevant, as it underscores the importance of restoring mucosal microenvironmental homeostasis, rather than focusing exclusively on downstream inflammatory pathways. From a translational perspective, these findings are particularly relevant because impairment of ASL homeostasis and MCC dysfunction represent central pathological features shared across multiple upper airway inflammatory diseases. By simultaneously combining direct anti-inflammatory activity with preservation of mucosal biophysical defense mechanisms, HMW-HA-based nasal formulations may provide broader therapeutic benefits than conventional saline-based nasal care strategies. Together with accumulating evidence supporting the safety, mucoadhesive properties, and reparative potential of intranasal HA, our results further strengthen the rationale for the clinical translation of physiologically optimized HA-based nasal therapeutics in chronic inflammatory sinonasal disorders.

In summary, both *in vivo* and *ex vivo* mouse experiments demonstrated that sodium hyaluronate nasal spray provides superior protection to the nasal mucosa compared with conventional saline spray. This protective effect is mediated through the maintenance of ASL integrity and MCC function. Application of sodium hyaluronate nasal spray preserved the structural integrity of the mucosal surface liquid and enhanced MCC, reducing the likelihood of allergen exposure to the mucosa, thereby attenuating local and systemic inflammation and mitigating epithelial barrier disruption. From a translational perspective, these findings challenge the conventional reliance on saline irrigation and support the development of ASL-mimetic therapies. HA-based formulations may represent a new class of interventions that integrate hydration, mechanical protection, and indirect immunomodulation.

Nevertheless, several limitations should be acknowledged. First, the relatively small sample size (*n* = 6 per group), although standard for murine models and sufficient to detect the major effects observed for MCC function and mucus integrity, may still limit statistical robustness for subtle secondary endpoints. Second, while our findings support an association between HA treatment, preservation of ASL homeostasis, and sustained MCC function, the precise molecular and biophysical mechanisms underlying these protective effects were not directly investigated. In the present study, the protective effects of HA were primarily inferred from preservation of mucus layer thickness and delayed decline of MCC function observed in both *in vivo* and *ex vivo* experiments. Direct measurements of ASL rheology, osmolarity, hydration status, and ciliary beat frequency were not performed because of experimental and technical limitations. Therefore, the proposed mechanism should be interpreted with appropriate caution. Nevertheless, the combined *in vivo* and *ex vivo* findings consistently support the hypothesis that maintenance of the ASL microenvironment contributes substantially to the protective effects of HA. In particular, pathways related to epithelial ion transport, ciliary beat regulation, HA receptor signaling, mucus rheology, and ASL biophysical properties warrant further investigation. Third, the sodium hyaluronate nasal spray evaluated in this study consisted of a single concentration and defined molecular weight range; therefore, future studies should further explore dose–response relationships and molecular weight–dependent effects of HA, as well as its long-term safety. Finally, murine nasal physiology may not fully recapitulate the complexity of human sinonasal disease. Nonetheless, our findings support HA-mediated preservation of ASL integrity as a protective mechanism and highlight the translational potential of HA-based ASL-mimetic nasal formulations for upper airway inflammatory disorders, which may ultimately benefit patients with nasal diseases.

## Data Availability

The original contributions presented in the study are included in the article/Supplementary Material, further inquiries can be directed to the corresponding authors.
